# Supplementary material for: A problem of Epic proportion
Source: PLOS Digit Health. 2026 Mar 23;5(3):e0001143. doi: 10.1371/journal.pdig.0001143 (PMC13008087; doi:10.1371/journal.pdig.0001143)
Supplement: Attachment — Submitted filename: Detailed Response to Reviewers.docx [file pdig.0001143.s001.docx]

**Responses to Editorial Decision Letter on the paper titled:**

**A problem of Epic proportion**

**Manuscript# PDIG-D-25-01138**

**Section #1: Includes response to the Section Editor**

We would like to thank the section editor, and the reviewers for their positive feedback, comments, suggestions, and for giving us the opportunity to revise and resubmit the paper. We would like to revise the manuscript and re-submit based on the comments and suggestions for all participants involved. To ensure that all editor and reviewers’ concerns and suggestions have been addressed, the authors have detailed all adjustments to the manuscript using the blue color.

**Section #2: Includes our Point-to-Point responses to the Reviewers (grouped by reviewers).**

**Response to Reviewer #1’s Comments:**

**Reviewer #1’s comment 1:**

This manuscript provides a timely and comprehensive analysis of structural power in the U.S. EHR market. The manuscript is well written, conceptually rich, and raises important policy-relevant questions. The Introduction clearly and effectively articulates the overarching problems addressed in this study, and I found the stepwise transformation framework particularly engaging. I believe this work will be of interest to a broad audience in digital health.

I recommend acceptance of this manuscript, subject to minor editorial revisions. As part of the publication process, I encourage the authors to review the manuscript carefully and address the following editable points:
1. Introduction, line 15: There is a typographical error — “HITEC” should be corrected to “HITECH.”
2. Use of acronyms: Acronyms such as FHIR appear before their full terms are defined (e.g., FHIR first appears at line 109, while the full term is introduced later at line 157). Similarly, TEFCA appears at line 109, while the full term is defined at line 378. The full term should be introduced at its first mention.

**Authors' response 1:**

We sincerely thank the reviewer for their careful reading of the manuscript and for their generous and encouraging assessment of its contribution, clarity, and policy relevance. We appreciate the reviewer’s attention to detail. In response to the minor editorial points raised, we have corrected the typographical error in the Introduction by changing “HITEC” to “HITECH.” We have also revised the manuscript to ensure that all acronyms are defined at their first mention, including Fast Healthcare Interoperability Resources (FHIR) and the Trusted Exchange Framework and Common Agreement (TEFCA), and have used the acronyms consistently throughout the manuscript. These revisions improve clarity and readability while preserving the substance of the analysis.

**Response to Reviewer #2’s Comments:**

**Reviewer #2’s comment 1:**

This manuscript presents a timely and well-executed analysis of electronic health record (EHR) market concentration in the United States, with Epic Systems examined as a central case. The topic is highly relevant to the scope of PLOS Digital Health and addresses important questions related to interoperability, governance, and public accountability in digital health infrastructure.

The analytical approach is appropriate and rigorous. The authors effectively integrate quantitative market concentration metrics with qualitative synthesis of peer-reviewed literature, regulatory history, legal filings, and international case examples. The statistical analyses are sound, clearly described, and properly interpreted, and the conclusions are well supported by the presented evidence without overstatement.

Data sources are transparently cited and publicly available, and the Data Availability Statement accurately reflects the nature of the underlying data. Ethical and publication considerations, including ongoing litigation and the use of Epic-derived clinical data, are handled carefully and responsibly.

The manuscript is clearly written, well structured, and accessible to an interdisciplinary audience. Overall, this study makes a valuable contribution to the digital health and health policy literature, and I recommend acceptance without revision.

**Authors' response 1:**

We sincerely thank the reviewer for their careful reading of the manuscript and for their thoughtful and generous evaluation. We appreciate the reviewer’s recognition of the rigor of our analytical approach, the integration of quantitative and qualitative evidence, and the attention to ethical considerations, data transparency, and responsible treatment of ongoing litigation. We are grateful for the reviewer’s assessment that the manuscript is clearly written, methodologically sound, and well aligned with the scope of *PLOS Digital Health*. As no specific revisions were requested, we have not made changes in response to this review.

**Response to Reviewer #3’s Comments:**

**Reviewer #3’s comment 1:**

General Assessment: This paper presents an exceptionally timely and rigorous analysis of market concentration in the U.S. EHR sector. The authors effectively argue that Epic’s dominance is driven not merely by product superiority but by structural market forces, utilizing international case studies (Norway, Denmark, UK) to highlight how U.S.-specific market structures have facilitated this consolidation. The proposed policy reforms are well-reasoned and highly relevant to current legislative discussions.

To further strengthen the manuscript and ensure precision, a minor revision is required.

Specific Comments:
1. Clarification of Legal Terminology regarding Pending Litigation
Context: In the "Stakes" section (Line 873), the manuscript notes that "Federal litigation has documented oncology patients losing access...".
Comment: While the author likely refers to plaintiff complaints, the phrase "litigation has documented" risks misrepresenting unproven allegations as adjudicated facts. Since Particle Health v. Epic and CureIS v. Epic are ongoing, I recommend qualifying these statements with phrases such as "allegedly," "claims that," or "according to the filings" to strictly maintain the manuscript's legal precision and neutrality.

**Authors' response 1:**

We sincerely thank the reviewer for their thoughtful and careful reading of the manuscript and for their helpful guidance on maintaining legal precision and neutrality. We appreciate the reviewer’s recognition of the timeliness, rigor, and policy relevance of the analysis.

In response to the specific comment regarding pending litigation, we have revised the language in the *Stakes* section to explicitly attribute references to patient record access and data-sharing disruptions to plaintiff allegations and court filings, rather than presenting them as adjudicated facts. This clarification ensures that the manuscript accurately reflects the ongoing status of the litigation and maintains appropriate legal precision, consistent with the reviewer’s recommendation.

We hope that the answers and responses to the reviewer’s and editor’s comments are satisfactory.
